# Evaluating the potential and eligibility of conservation agriculture practices for carbon credits

**DOI:** 10.1038/s41598-024-59262-6

**Published:** 2024-04-22

**Authors:** Adeeth A. G. Cariappa, Noufa C. Konath, Tek B. Sapkota, Vijesh V. Krishna

**Affiliations:** Sustainable Agri-Food Systems (SAS) Program, International Maize and Wheat Improvement Center (CIMMYT), ICRISAT Campus, Hyderabad, India

**Keywords:** Climate change, Environmental social sciences, Climate-change mitigation, Climate-change policy, Environmental economics

## Abstract

Carbon credits, a voluntary market mechanism to reduce greenhouse gas (GHG) emissions, can incentivize climate action. We evaluate the potential and eligibility of Conservation Agriculture (CA) practices for carbon credit generation in India under Verra's VM0042 methodology. Using farmer surveys and remote sensing data, we assess the eligibility based on the following conditions: Additionality Condition (GHG emission reductions to exceed legal requirements and the weighted mean adoption rate to be < 20% of area in the baseline), Yield Penalty Condition (no > 5% decrease in crop yields), and Quantitative Adjustment Condition (reduction in chemical fertilizer use by > 5%). Our analysis shows that CA has the potential to increase farmers’ carbon credit earnings by USD 18/ha and USD 30/ha in Bihar and Punjab, respectively. Punjab's ban on crop residue burning and the fact that > 20% of the area unburned limits the full economic realization of CA through carbon markets, decreasing potential income to USD 16/ha. A 60% increase in carbon prices from the current norm (USD 25) is required to encourage wider adoption of CA. Zero tillage of wheat in both Punjab and Bihar and reduction of nitrogen fertilizer overuse in Punjab fulfil all the conditions and are eligible for carbon farming projects.

## Introduction

As climate change escalates from ‘global warming’ to ‘global boiling,’ the urgency to act intensifies^1^.The imperative for climate action to curb greenhouse gas (GHG) emissions, once considered a luxury, has now become non-negotiable. To minimize the impacts of climate change, it is crucial to limit the temperature increase to below 2 °C above pre-industrial levels, while pursuing efforts to limit it to 1.5 °C^2^. The food production system, which is a component of agrifood systems, is at the core of this complex challenge, being a significant contributor to climate change and a victim of its impact^3^. On one hand, the agrifood sector is highly vulnerable, and small-scale farmers in the Global South are especially at risk as current efforts to adapt to climate change are not sufficient^4^. On the other, any immediate and deep reduction in emissions cannot be achieved without including the agrifood sector, as it accounts for nearly a third of the world's GHG emissions^5^.

While the developed nations are historically held responsible for a large share of the GHG emissions, the landscape is undergoing a seismic shift. Over the last two decades, despite having lower per-capita emissions than developed countries, China and India have witnessed a surge in emissions owing to their rapid economic development, calling for urgent climate action^6^. While there exists a line of argumentation that climate change mitigation should be pursued in low- and middle-income countries where it is most cost-effective^7^, such arguments are often contested on the grounds of fairness and equality, creating a hostile negotiating environment in international climate summits^8^.

Against these challenges, carbon credits have emerged as a promising voluntary market mechanism to reduce GHG emissions from production systems. A set of sustainable agricultural practices under the umbrella of carbon farming are promoted to enhance the soil’s ability to capture carbon, thereby decreasing the release of GHGs into the atmosphere^9^. Farmers, by adopting sustainable agricultural practices such as reduced tillage or optimized fertilizer use, can generate these credits and sell them in the market. This development could address the critical concern of sustainably transforming the agricultural systems without harming farmers’ economy.

Responding to the increasing significance of carbon markets, over 50 agricultural projects are currently active in India, claiming to contribute to an annual reduction of 37 million tonnes of carbon dioxide equivalent (CO_2_e) emissions^10^. Alongside international voluntary markets, national governments like India are establishing their own carbon markets. India's forthcoming compliance and voluntary carbon markets, and the green credit trading scheme will target eight key sectors, with a special emphasis on ‘Sustainable Agriculture based Green Credit’ to promote natural and regenerative farming, improve soil health, enhance food nutrition, and increase agricultural productivity^11–13^.

For carbon markets to be effective and credible, robust standards and stringent regulations are essential. Verra, a leading carbon credit certifier, requires project developers to adhere to specific standards and methodologies to earn verified carbon units (VCUs), or carbon credits^14^. This includes "VM0042: Methodology for Improved Agriculture Land Management, v2.0" tailored for agricultural projects which is active since 30 May 2023. Each carbon credit represents 1 tonne CO_2_e of reduced or sequestered GHG emissions. The VM0042 methodology provides a framework to measure, monitor, report, validate and verify agricultural carbon credits, ensuring their quality and contribution to climate change mitigation under Verra's governance^15^.

Despite the potential benefits of carbon credits, significant uncertainties persist in project execution, fear of greenwashing, risk of nonequivalent credit creation, emissions measurement, inflated baselines, especially in Reducing Emissions from Deforestation and Forest Degradation (REDD+) projects and the limited feasibility of including all mitigation strategies in carbon farming^16–20^. For example, evidence suggests that REDD+ projects have not significantly curtailed deforestation rates, and in instances where reductions have occurred, they tend to be markedly less than the levels claimed^18^. Therefore, the strategic blueprint for carbon projects must be rooted in systematic, scientific planning to avoid these potential pitfalls.

Among the different practices recommended for carbon capture, Conservation Agriculture (CA) emerges as a viable candidate. There is a history of research-and-development (R&D) projects focusing on CA in South Asia, spanning > 2 decades. Characterized by minimal soil disruption (through the adoption of zero/minimal tillage technologies), consistent soil cover, and crop rotation, CA not only reduces GHG emissions and sequesters carbon but also enhances crop yields^21–27^. Implementing only 3 practices, efficient fertilizer use, zero-tillage, and improved rice-water management could achieve > 50% of India's technical abatement potential, amounting to 85.5 million tonnes of CO_2_e annually^28^.

Using International Maize and Wheat Improvement Center's (CIMMYT) 2021 survey and remote sensing data, we aim to quantify the carbon credit generation potential of CA and estimate the additional income to farmers. Further, we assess the feasibility of CA practices for carbon credit generation under Verra’s VM0042 methodology in the wheat growing season (rabi) of India. We aim to empirically assess the following hypotheses, based on the applicability conditions for a carbon farming project.

### H1. Additionality

*H1.1. Regulatory surplus condition* The GHG emissions reductions must go above and beyond any legal requirements.

*H1.2. Adoption condition* The weighted mean adoption rate of sustainable agriculture practices is <20% before the project start date.

*H2. Yield penalty condition* The adoption of these practices does not reduce crop yield by > 5%.

*H3. Quantitative adjustment condition* Reduction in fertilizer usage exceeds 5% of the recommended dose for wheat in the region.

## Results

### The potential of CA to reduce GHG emissions through carbon credits in India

Boasting one of the world's largest agricultural sectors, India grapples with the dual challenge of mitigating GHG emissions while ensuring food security for its vast population. The emissions from crop residue burning alone have surged by 75% from 19,340 Gigagram (Gg) per annum in 2011 to an alarming 33,834 Gg in 2020^29^*.* Declining groundwater levels, land degradation, and excessive use of chemical fertilizers are exacerbating emission surges, while sustainably increasing food production amid these depleting resources poses a significant challenge for governments and R&D organizations.

**Figure 1:**
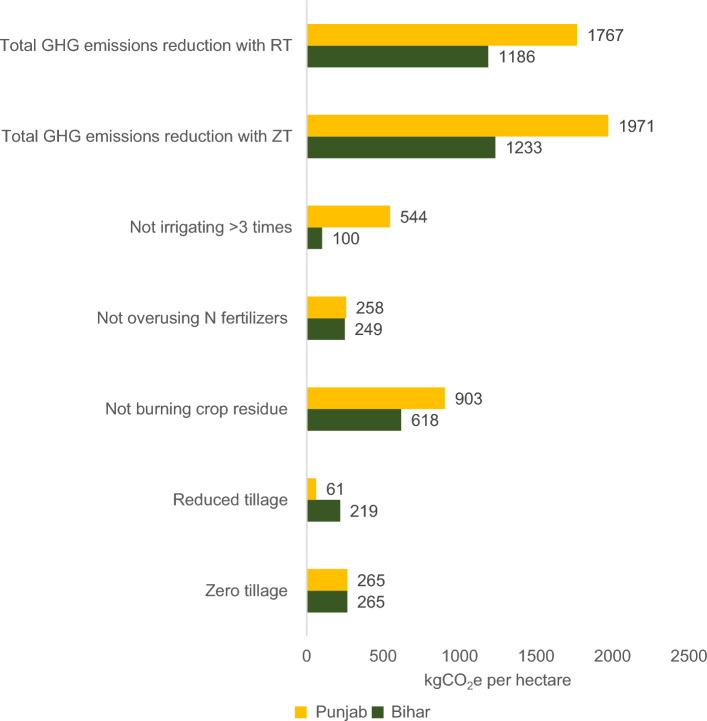
GHG emissions reduction from CA practices in wheat production. *Note*: Both Punjab and Bihar emissions have been ‘Winsorised' at a 5% level to mitigate the impact of outliers, ensuring more robust and reliable statistical analyses. Each of these values are propensity score matching (PSM) estimates of mean differences between adopters and non-adopters of specific CA practice (see note of Table 2). Zero-tillage is not practiced by the sampled households in Bihar. We assume a similar level of GHG emissions reduction in Bihar as Punjab at 265 kgCO_2_e per hectare. *Source*: Authors estimation based on CIMMYT Punjab Household Survey 2021 and CIMMYT Bihar Household Survey 2021.

We found that, through the adoption of CA practices such as zero/reduced tillage, cessation of residue burning, reduced nitrogen fertilizer overuse, and decreased irrigations in wheat production season, farmers can reduce GHG emissions by 1.23 to 1.97 tonnes of CO_2_e per hectare of land respectively in Bihar and Punjab States of the Indo Gangetic Plains (IGP) (Fig. 1). It translates to the creation of 1.23 to 1.97 carbon credits per hectare respectively. As per our discussion with carbon companies, the price of one carbon credit varies between USD 2–6 in marketplaces, and it can go up to USD 15–25 in over-the-counter deals. At the high end (at USD 25 per credit), the total value of these carbon credits in the wheat season will be around INR 2400 (USD 31) in Bihar and INR 3800 (USD 49) in Punjab (Table 1). If we consider the average operational land holdings (0.6 ha in Bihar and 4.9 ha in Punjab) and the proportion of revenue from carbon credit sales that would reach the farmer (60% of total revenue generated by the sales of carbon credits), the scenario changes. A typical farmer in Bihar would receive around INR 900 (USD 11) (equivalent to 2% of their bi-annual income), while a farmer in Punjab would receive around INR 11,000 (USD 145) (7% of their bi-annual income) during the wheat growing season.

**Table 1:** Carbon credits generated from wheat season and its value. *Source*: Authors estimation based on CIMMYT Punjab Household Survey 2021 and CIMMYT Bihar Household Survey 2021.

	Bihar	Punjab
Carbon credits generated (numbers)	1.23	1.97
Value of carbon credits per hectare @ USD 25 per credit	INR 2423 (~ USD 31)	INR 3873 (~ USD 49)
60% per-hectare value of carbon credits that reaches farmer in wheat season	INR 1454 (~ USD 18)	INR 2324 (~ USD 30)
Additional revenue for typical farmer (cultivating in 0.6 ha in Bihar and 4.9 ha in Punjab, under assumption of full adoption)	INR 872 (~ USD 11)	INR 11,386 (~ USD 145)
Increase in revenue from carbon credits as % of farmers’ income	1.93	7.11

*Carbon Credit Conversion*: 1 carbon credit is equivalent to mitigating 1000 kgCO_2_e emissions.

*Carbon Credit Pricing*: The carbon credit value is set at USD 25 per credit. This is based on discussions with carbon companies and expectations of rising credit prices due to increasing demand for credible credits.

*Official exchange rate for 2022*: 1 USD = INR 78.6 is used for conversion (https://wdi.worldbank.org/table/4.16).

*Revenue Sharing*: Carbon credit companies distribute around 70% of *net* revenue to farmers. We assume, after deducting 10% for registration and other costs, 60% of the gross revenue might reach farmers.

*Typical Farmer*: The typical farmer in Bihar and Punjab owns 0.6 and 4.9 hectares of land, respectively. Income data is obtained from the Situation Assessment Survey of Agricultural Households, conducted by the National Statistical Office, Ministry of Statistics and Programme Implementation, Government of India during the 77th round (January 2019–December 2019) with reference to the agricultural year July 2018-June 2019. Average monthly income in Punjab and Bihar is INR 26,701 (USD 322) and INR 7542 (USD 91), respectively.

*Assumption of full adoption*: It is assumed that all farmers in the carbon farming project will adopt the Conservation Agriculture (CA) practices in their whole wheat area.

As anticipated, Punjab's farmers stand to generate a higher carbon credit yield per hectare than Bihar. It is largely driven by larger land holdings and higher base effects—especially the higher potential emissions reductions from not burning crop residues and optimizing winter irrigation in Punjab (Fig. 1).

We evaluate the effectiveness of carbon credits in promoting CA in Punjab, referencing a recent randomized controlled trial (RCT) conducted in the same region where farmers were paid to avoid burning crop residues, based on the principles of Payments for Ecosystem Services (PES). This RCT, conducted across 171 villages had three groups: a control group without a contract, a standard PES group with post-verification payment, and an upfront PES group receiving a partial initial payment (25 or 50%) with the balance post-verification^30^. Remarkably, the treatment arm offering 25–50% upfront payment of the total USD 26 per hectare emerged as the most cost-effective, leading to significant reduction in residue burning. The figure, USD 26 per hectare, was determined by researchers based on the marginal cost of crop residue management. Our analysis suggests that carbon credits yield approximately USD 30 per hectare in Punjab. Allocating 25–50% of this as an upfront payment could notably enhance CA adoption. While CA practices have typically been promoted for their profitability, adding carbon credits as a supplementary incentive could further bolster these initiatives.

### Evaluation of eligibility of various CA components under VM0042 carbon projects

Having established the potential of CA to generate carbon credits, we will now assess whether CA practices meet all the eligibility criteria for inclusion in carbon farming projects.

### H1.1. Regulatory surplus condition: prohibition by law or regulation

According to the VM0042 methodology, R&D activities are ineligible for inclusion in carbon farming projects and subsequent carbon credits if they are already prohibited by law. This means that activities such as abstaining from burning crop residues would be ineligible in states like Punjab and Haryana, where crop residue burning is legally banned^31^.

When cessation of residue burning is excluded from our calculations for CA (Supplementary Table 3), the creation of carbon credits will reduce to 1.1 tonnes of CO_2_e per hectare in Punjab, reducing the total value of these carbon credits in the wheat season from USD 49 to USD 27. Farmer revenue (at 60% allocation rate) decreases from USD 30 to USD 16 per hectare. Now, the estimated benefits from carbon markets can be considered insufficient to promote CA, especially in Punjab. A recent RCT shows that crop residue burning in Punjab can be significantly reduced with an advance payment of USD 26 per hectare^30^. The prices for carbon credits would have to increase by 60% of the current value to meet farmers’ expectations derived from the RCT. These results make it clear that a significant rise in carbon credit pricing is required and the low carbon prices could fail to incentivize farmers to adopt and pose the risk of discontinuation of CA practices, threatening the permanence condition of carbon credits. This reduction underscores the challenge of harmonizing environmental regulations and incentives with on-the-ground agricultural practices, thereby diminishing the effectiveness of carbon markets.

These results reveal the complexity of criteria for practice inclusion in carbon markets and their impact on carbon farming project design and implementation. They emphasize the need to comprehend local contexts, practices, and legal constraints, and recognize the dual potential for environmental and economic benefits for farmers.

### H1.2. Adoption condition: < 20% adoption rate

**Figure 2:**
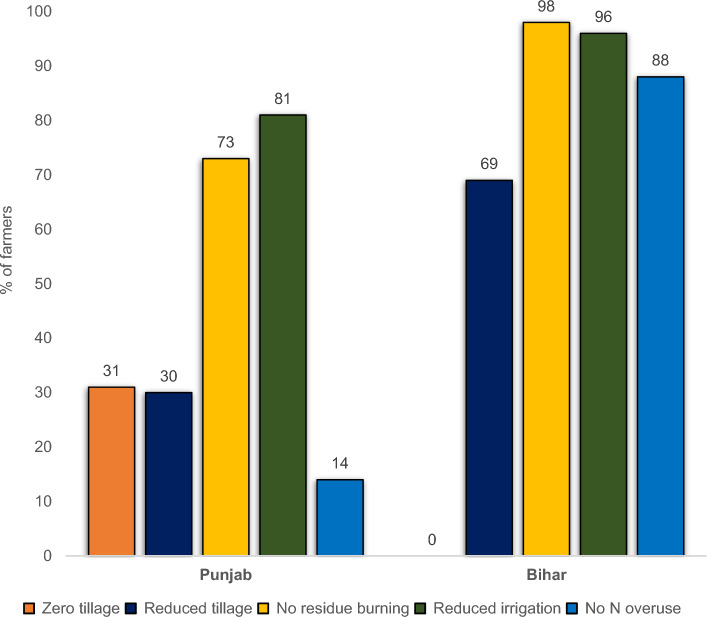
Adoption of sustainable agriculture practices in Punjab and Bihar. *Note:* The adoption of sustainable agricultural practices, when measured as a percentage of area, corresponds closely to the percentage of farmers adopting these practices. For example, the adoption of zero-tillage and reduced tillage in Punjab accounts for 32% and 28% of the total wheat-cultivated area, respectively. *Source*: Authors’ estimation based on CIMMYT Punjab Household Survey 2021 and CIMMYT Bihar Household Survey 2021.

Our survey data reveals intriguing patterns in CA adoption in both Punjab and Bihar (Fig. 2). Approximately 30% of Punjab farmers have adopted zero or reduced tillage, which signifies that it has become a “common practice”. If this is the case in all districts of Punjab, this tillage technology alone does not qualify for carbon farming under VM0042. Similarly, only about 27% of respondents in Punjab reported burning crop residues, implying that 73% do not engage in this practice. Thus, not burning rice residues is a “common practice”, and consequently, it is ineligible for inclusion in carbon farming projects. Further, only a minority (19%) of Punjab farmers irrigate their fields > 3 times, indicating that the majority already practice reduced irrigation, making this practice also ineligible for carbon farming projects. Finally, a substantial (86%) share of Punjab farmers overuse nitrogen-based fertilizers (Urea and Diammonium phosphate (DAP)), suggesting that interventions to reduce chemical fertilizer use would be eligible for VM0042 carbon farming projects.

While Verra's VM0042 mandates the use of percentage of cultivated area to assess “common practice” condition, practical difficulties arise in estimating the percentage of area for each management practice due to the lack of comprehensive data from all plots cultivated by farmers in Bihar, and for some practices in Punjab. Consequently, the next best metric, the percentage of farmers who have adopted these practices, was used. Since the adoption measured as a percentage of area corresponds closely to the percentage of farmers adopting these practices, this approach does not compromise the accuracy of the common practice analysis. For instance, the adoption rates in terms of area and farmers for zero tillage are 32% and 31%, for reduced tillage 28% and 30%, for no residue burning 76% and 73%, and for reduced irrigation 71% and 81%, respectively.

In contrast to Punjab, Bihar farmers are already practicing less intensive agriculture, potentially driven by financial constraints and lack of market access, rather than conscious choice. None of the CA practices examined (reduced tillage (tilling the land < 2 times), avoiding residue burning, limited irrigation, and restrained use of nitrogen fertilizers) qualify for carbon farming projects in Bihar. However, no farmers in Bihar were found to practice zero-tillage, unlike in Punjab, suggesting that zero-tillage could be a potential intervention in Bihar to be covered under carbon markets.

**Figure 3:**
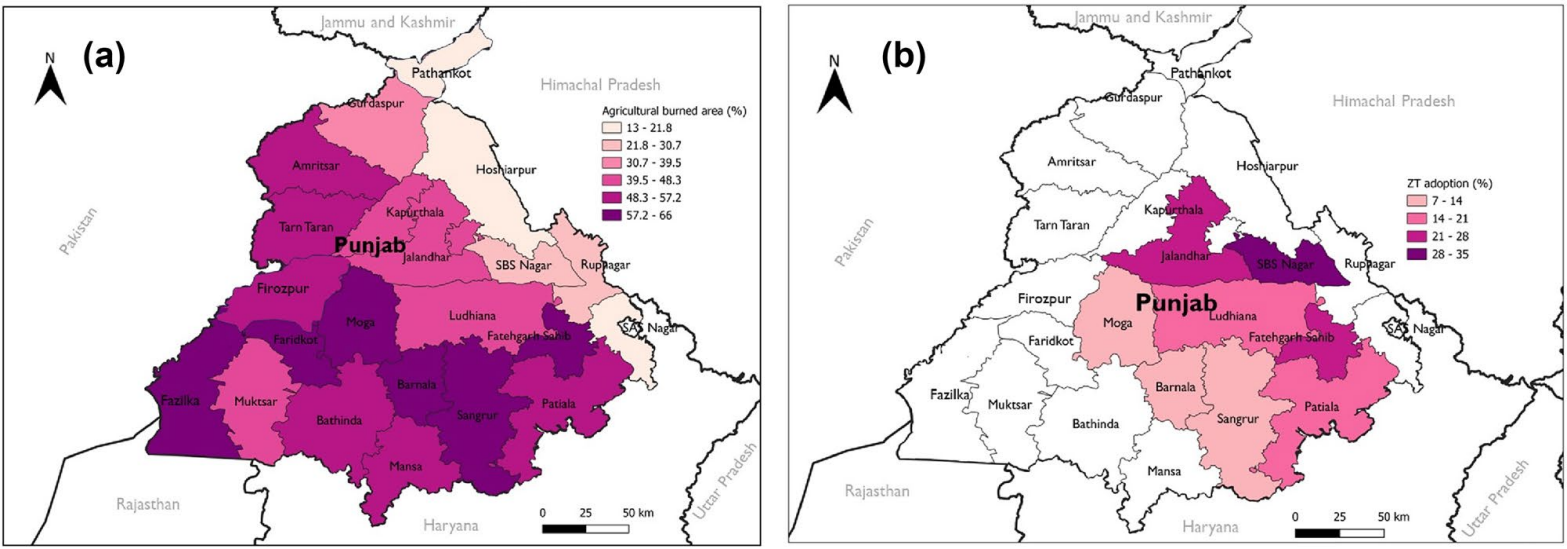
(**a**) Crop residue burning in Punjab during 2022. (**b**). Adoption of zero-tillage (ZT) in wheat in Punjab during 2022. *Source*: Authors estimation based on remote sensing data. Map created using QGIS Desktop 3.30.3, a free and open-source Geographic Information System, available at: https://qgis.org. The base maps were derived from shapefiles obtained from the open source Administrative Boundary Database of the Survey of India (https://onlinemaps.surveyofindia.gov.in/Digital_Product_Show.aspx).

The survey data reveals that zero-tillage and abstaining from burning rice residues do not qualify for VM0042 carbon farming projects in Punjab due to their adoption rates exceeding 20%. This finding is surprising considering that stubble burning in Punjab and Haryana is cited as a major contributor to winter air pollution in New Delhi^30,32^. Especially in Punjab, since area under residue burning in the past decade has increased by 46% while it decreased by 11% in Haryana^29^. To investigate this further, we utilized remote sensing data, which provided interesting results (Fig. Fig. 3a,b). In Punjab, crop residues in 45% of the cultivated area was burnt in 2022 (Supplementary Table 1) with some districts reporting rates as high as 66%. Farmers often underreport residue burning, with satellite data revealing a higher prevalence compared to face-to-face interviews in the surveyed regions^33^. This phenomenon underscores the need for more accurate and comprehensive data collection methods when assessing agricultural practices that cause negative externalities. Despite the higher burning rates observed, over 34% of the area remains unburned. This means that not burning is still a “common practice” making it ineligible for carbon farming projects in Punjab.

Remote sensing data was also employed to assess zero-tillage adoption in Punjab. Among the eight districts for which data was available, four had a zero-tillage adoption rate of < 20% (Fig. 3b). Thus, zero-tillage is not a “common practice” in all districts, making it a potential project intervention at least in a few districts. We also identified a strong negative relationship between the adoption of zero-tillage and stubble burning (Fig. 3a,b), suggesting that regions with high residue burning and low zero-tillage adoption could be targeted by carbon farming project developers. Such a strategy could lead to greater GHG emissions reduction, generating more carbon credits and income for farmers, which might incentivize the continued practice of CA.

**Figure 4:**
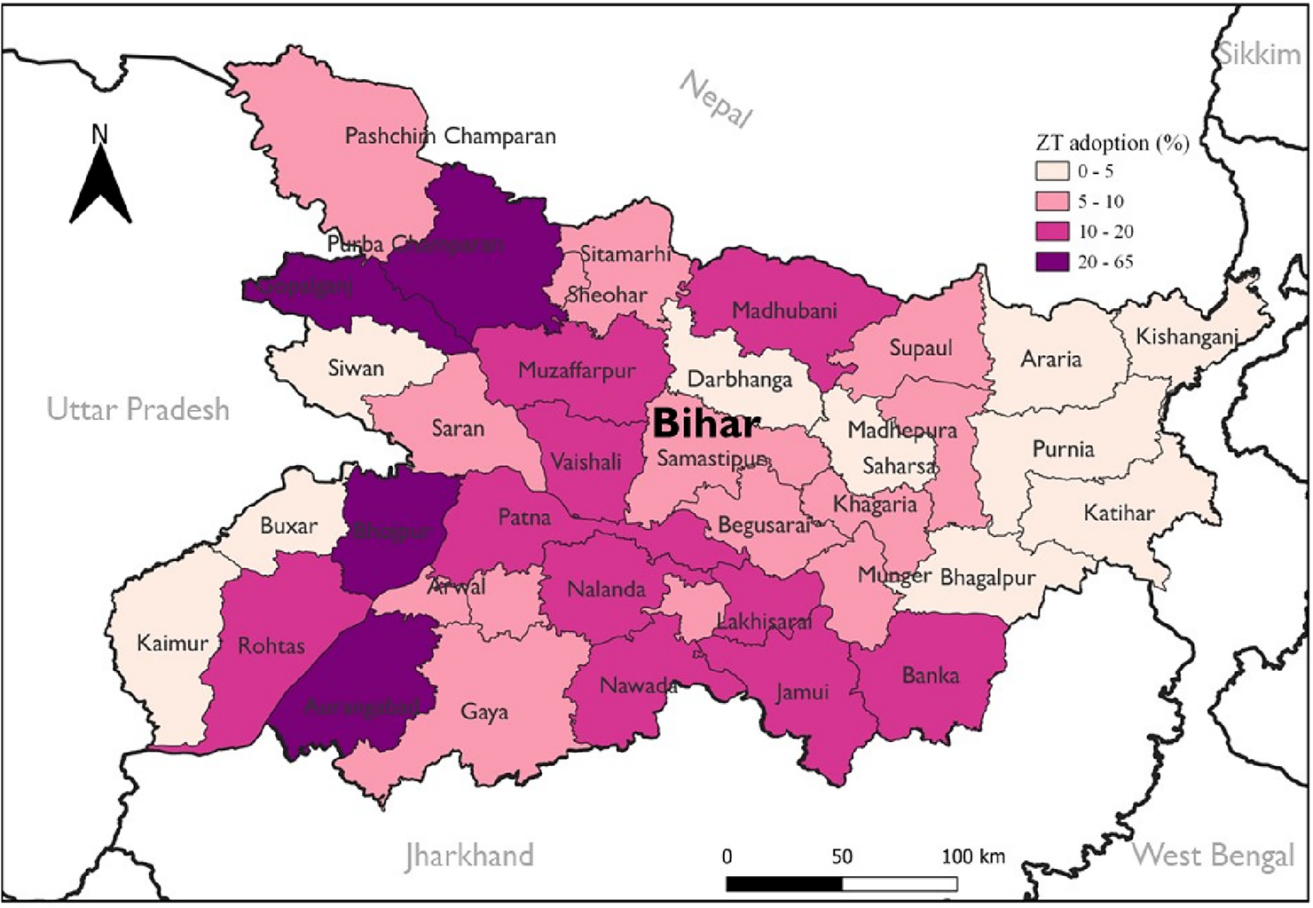
Adoption of zero-tillage (ZT) in wheat in Bihar during 2021. *Source*: CIMMYT Key Informant Survey 2021. Map created using QGIS Desktop 3.30.3, a free and open-source Geographic Information System, available at: https://qgis.org. The base maps were derived from shapefiles obtained from the open source Administrative Boundary Database of the Survey of India (https://onlinemaps.surveyofindia.gov.in/Digital_Product_Show.aspx).

Turning our attention to Bihar, we noted earlier that zero-tillage could be a potential intervention under VM0042, as none of the farmers in the sample was practicing zero-tillage. To validate this point, we utilized another dataset, the CIMMYT Key Informant Survey 2021, which collected data from all districts of Bihar. Out of the 38 districts, 34 had a zero-tillage adoption rate of < 20% in wheat (Fig. 4). Thus, zero-tillage is not a “common practice” and can be eligible for carbon projects. As zero-tillage adoption is currently low in Bihar, it could be coupled with other practices like water or nutrient management, so the weighted mean adoption rate remains < 20%, and a greater number of carbon credits could be generated.

### H2. Yield penalty condition: yield reduction is < 5%

It is crucial to ensure that CA practices in carbon farming projects do not lead to notable yield reductions or jeopardize food security, particularly in low- and middle-income countries. To quantify the yield penalty and isolate the effects, we applied a propensity score matching (PSM) technique. This methodology is well-regarded for estimating the impacts of interventions in the absence of experimental data^34,35^. Interestingly, after controlling for soil type, soil fertility, sowing date, land preparation, water management, nutrient management, farmer characteristics and district dummies, we found no evidence of a significant yield penalty when farmers adopt CA practices such as refraining from burning residue, zero/reduced tillage, reducing nitrogen fertilizer use, and reducing irrigation frequency (Table 2). Our findings align with existing literature, either indicating a lack of yield penalty or suggesting an increase in wheat yields^22–24,26,36^. These findings suggest that the identified CA practices fulfil the yield penalty condition and thus qualify for inclusion in carbon farming projects in India.

**Table 2:** Effect of sustainable intensification practices on wheat yield and GHG emissions in Punjab and Bihar. *Source*: Authors estimation based on CIMMYT Punjab Household Survey 2021 (n = 983) and CIMMYT Bihar Household Survey 2021 (n = 831).

	Outcome variables			
	GHG emissions (kgCO_2_e/hectare)		Marginal effects on Yield (% change)	
	Bihar	Punjab	Bihar	Punjab
Zero-tillage, ZT (1/0)		− 265.5** (107.1)		− 0.3
Reduced tillage, RT (1/0)	− 218.7*** (34.2)	− 61.4 (101.1)	− 8.0	− 0.6
Not burning crop residue (1/0)	617.7*** (168.7)	903.1*** (91.8)	23.4	2.8***
Not overusing nitrogen fertilizers (1/0)	249.4** (109.4)	257.9 (178.4)	− 3.4	− 1.4
Not irrigating > 3 times (1/0)	100.3** (45.0)	544.4*** (180.7)	− 1.8	0.6

Standard errors of the estimated effects in Propensity Score Matching (PSM) approach are shown in parentheses.

**p < 0.05, ***p < 0.01.

PSM was estimated using the following variables:

ZT/RT in Punjab: household characteristics (age, education, caste, household size), landholding, soil characteristics (soil type, fertility), water logging in plot and district dummies.

RT in Bihar: household characteristics (caste, household size), landholding, land tenure status, soil characteristics (soil type, fertility), plot characteristics (erosion problems, salinity/sodicity problem, water logging), wheat sowing month, and district dummies.

Not burning crop residue in Punjab: household characteristics (age, education, caste, household size), landholding, soil characteristics (soil type, fertility), number of cattle and buffaloes owned, tillage, and district dummies.

Not burning crop residue in Bihar: household characteristics (caste, household size), landholding, land tenure status, soil characteristics (soil type, fertility), plot characteristics (erosion problems, salinity/sodicity problem), wheat sowing month, number of cattle and buffaloes owned, tillage and district dummies.

Not overusing nitrogen fertilizers in Punjab: household characteristics (age, education, caste, household size), landholding, soil characteristics (soil type, fertility), water logging in plot, manure use, mulching and district dummies.

Not overusing nitrogen fertilizers in Bihar: household characteristics (caste, household size), landholding, land tenure status, soil characteristics (soil type, fertility), plot characteristics (slope, erosion problems, salinity/sodicity problem, water logging), wheat sowing month, manure use, irrigation, and district dummies.

Not irrigating > 3 times in Punjab: household characteristics (age, education, caste, household size), landholding, soil characteristics (soil type, fertility), tillage, mulching, manure use, and district dummies.

Not irrigating > 3 times in Bihar: household characteristics (caste, household size), landholding, land tenure status, soil characteristics (soil type, fertility), plot characteristics (salinity/sodicity problem, water logging), wheat sowing month, tillage, land levelling and district dummies.

The broader implications of these results are profound. Not only do they confirm the possibility of integrating sustainable practices into agricultural processes without compromising yield, but they also highlight the potential for these practices to contribute to climate change mitigation. This emphasizes the broader value of carbon farming initiatives, which, beyond their core objective of facilitating carbon certificate trading under protocols like VM0042, offer the added advantages of promoting environmental sustainability, maintaining food security, and potentially increasing farmers' incomes.

### H3. Quantitative adjustment condition: reduction in fertilizer use is > 5% of the recommended dose for wheat

Earlier, we observed that most farmers overuse nitrogen fertilizers in Punjab. With the practice of reducing nitrogen fertilizer rates meeting adoption and yield penalty conditions, we now proceed to test whether there is scope for a > 5% reduction in nitrogen fertilizer use.

To this end, we compared farmers' fertilizer use with the recommended dose. According to the Punjab Agriculture University (PAU), wheat crops require 271.7 kg/hectare of urea and 135.85 kg/hectare of DAP. Our findings reveal that farmers apply 16% more urea and 69% more DAP than the recommended dose (Supplementary Table 4). In terms of nitrogen, we found that farmers apply 48% more than the recommended dose of 123.5 kg/hectare. This pattern of nitrogen overuse has been identified in the literature^37–39^. Consequently, there is a scope for a quantitative adjustment in the application of fertilizers exceeding 5%. Nevertheless, stringent monitoring of nitrogen application practices is imperative. The challenge lies in accurately tracking farmers' nitrogen fertilizer usage. Ineffective monitoring can compromise the integrity of the carbon credits generated, rendering them unattractive to potential buyers. A market saturated with 'bad lemons' or low-quality carbon credits^40^ could emerge because of unscientific practices and measurement, poor monitoring, and faulty validation and verification processes. Thus, although the reduction of nitrogen fertilizer overuse is a qualifying activity for carbon farming projects, the success of these initiatives hinges on a robust Monitoring, Reporting, and Verification (MRV) system to ensure credibility and effectiveness.

The broader implications of this finding extend beyond GHG emissions and carbon credits. A reduction in fertilizer overuse can decrease cultivation costs for farmers and increase profitability. At the macro level, it can lessen the subsidy burden on the government. The fertilizer subsidy costs over USD 30 billion to the government of India. This underscores the potential of carbon farming projects to contribute to both environmental sustainability and economic efficiency.

### Discussion: implications of carbon farming in Indian agriculture

Carbon credit projects offer a promising market-based approach to incentivizing farmers to adopt sustainable agricultural practices. If the entire area under wheat in Bihar and Punjab states were covered within a carbon credit project, it could mitigate approximately 2.7 and 7.0 MtCO_2_e GHG emissions, respectively, equivalent to the annual emissions produced by 1.9 million gasoline-powered passenger vehicles^41^. Even in a less optimistic scenario where only one-third of the wheat area is covered in a carbon project, around 900,000 carbon credits would be generated in Bihar and 2.3 million in Punjab, which translates to USD 14 million and USD 34 million for the two states (farmers’ share), respectively. These figures exceed the USD 33 million budget allocated to Punjab under the Government of India's "Crop Residue Management Scheme" in the fiscal year 2022–23^42^, underscoring the significant financial incentives that carbon credit projects can offer to farmers.

However, there are several challenges that carbon projects or markets may encounter in India. One significant challenge is the strictness of Verra's standards and methodology, which makes it impossible to include heavily polluting agricultural practices in carbon farming projects. For example, the practice of not burning crop residue is deemed ineligible because it is legally banned and > 20% of farmers in Punjab are already not burning it, thus it is not considered additional. Verra’s VM0042 adopts the 20% threshold of common practice from the CDM methodological tool “Common Practice”^43^. The underlying notion for these cutoffs is that technologies/practices are still in the early stages of adoption and require support/incentives, such as carbon credits, to facilitate wider diffusion^44,45^. A comprehensive review and analysis of various diffusion of innovation theories and cutoffs from literature demonstrates the practicality of this 20% cutoff for identifying common practices^44^. A detailed discussion on the rationale behind the cutoffs, including diffusion theories, common practice metrics such as market penetration rates, and other additionality tests (which are beyond the scope of this paper), is available in the literature^44–47^.

It may be argued that exceptions could be made for heavily polluting practices such as crop residue burning, which continues to rise despite substantial policy and governmental interventions. For instance, in 2020, GHG emissions from residue burning reached 75% of the levels recorded in 2011, with a 240% increase in central India and a 46% increase in Punjab, despite legal bans^29^. This indicates that the applicable legal requirements are systematically not enforced, and that non-compliance is widespread which makes residue burning pass the first additionality test of demonstrating regulatory surplus as per the CDM tool for the “demonstration and assessment of additionality”^48^. However, VM0042 does not explicitly address the acceptability of practices in cases of poor enforcement. Assuming applicability to VM0042, the subsequent step in assessing additionality involves barrier analysis. The project developers must identify institutional barriers that would prevent the implementation of a change in pre-existing ALM practices to demonstrate additionality. If it is done successfully, the next step is the common practice analysis where the project developers demonstrate that the project activities are adopted on < 20% area before the project began. In exceptional cases, such as those of residue burning, the 20% threshold could be relaxed, and additionality can be assessed solely based on other additionality tests like the “barrier analysis” (does the project otherwise face significant or prohibitive barriers?)^45^. Emissions cutoffs can standardize exceptions, allowing practices exceeding a certain GHG emission threshold (e.g., 4–5 tCO2e/ha) to qualify for alternative additionality tests if they do not meet the common practice criterion.

If such an exception is made, we find that, using the same dataset, there is an 85% increase in emissions reduced, carbon credits generated, and the additional income that farmers receive from the sales of carbon credits. A typical farmer in Punjab would earn an additional income of around INR 11,000 per wheat season compared to INR 6,000 (Supplementary Table 3). Given that enforcing agricultural regulations in developing countries like India is often a complex political-economic challenge^49^, market-based instruments like carbon credits could provide a viable alternative. If CA practices, such as zero-tillage, can reduce burning at a faster pace and scale than current bans, not burning residue must be brought under carbon farming projects. However, for this approach to be effective, the ongoing carbon farming projects in India need to demonstrate success by generating high-quality carbon credits. Otherwise, the credibility of agriculture-based carbon credits will be compromised, leading to a market dominated by low-quality credits, which would discourage buyers from investing in agriculture-based carbon credits. Considering the huge negative environmental consequences associated with practices such as crop residue burning, which extend to regions as distant as New Delhi, we argue that Verra must reconsider and potentially ease the eligibility criteria when the negative externality associated with the practices is high, such as the case of residue burning.

Next, current carbon markets, with their limited incentives and reach, inadvertently create perverse incentives, disproportionately benefiting major polluters without significantly curbing pollution. However, as carbon markets grow and prices rise, there is a risk that farmers might intensify harmful practices like burning or ploughing to inflate baseline emissions (create higher 'starting point' for emissions), maximizing future carbon credit earnings.

The other challenge is the price of carbon credits. The level of incentives per unit of land might be insufficient to ensure adoption and continued compliance. Our estimates suggest that Punjab farmers could get an additional income of around USD 16 per hectare from carbon credits (excluding emissions reduction from crop residue burning, as it is ineligible for carbon farming due to the ban) while a recent RCT suggests it might require USD 26 for the farmers to adopt no burning practice^30^. Therefore, the price of carbon credit must at least rise by 60% to ensure continued farmer participation in carbon credit projects. To support the nascent carbon market for agricultural carbon credits, the Indian government could repurpose a portion of its existing fertilizer or electricity subsidy programs to provide farmers with deficiency payment (the difference between the market price and the base minimum price) until the market matures and prices rise. The base price for carbon credits might align with the recommendation of the High-Level Commission on Carbon Prices for developing countries (USD 40–80/tCO_2_e by 2020 and USD 50–100/tCO_2_e by 2030)^50^.

Despite these concerns, Bihar stands to gain more substantial social benefits due to its larger population of farming households living below the poverty line^22^. Bihar, in particular, has the potential to scale up these practices with 16 million operational holdings across approximately 7 million hectares, in contrast to Punjab, which has 1 million holdings covering 4 million hectares^51^.

To achieve these potential benefits, carbon credit projects must be carefully planned, designed, monitored, and implemented. This includes selecting the right interventions and project areas, engaging with farmers effectively, and ensuring robust monitoring and implementation mechanisms. To ensure successful implementation, an extensive extension network is crucial, such as partnering with local NGOs. In certain carbon projects in India, the strategy involves engaging untrained villagers to promote technologies and monitor adoption. However, this approach carries the risk of potential backfiring and threatening the overall adoption of project activities. Successfully implemented carbon credit projects can serve as a compelling market instrument to incentivize farmers to adopt and continue CA practices. This would create a win–win scenario for all stakeholders involved, including farmers, carbon credit businesses, corporate customers, the government, and the whole economy. Farmers would enjoy an additional income source, private sectors would engage in employment-generating activities, the government would realize cost savings, and economic growth would be stimulated through the demand generated by these activities. All these hinges on the right implementation of the project activities and the generation of credible carbon credits.

## Methods

This section provides a brief overview of the VM0042 methodological requirements to meet additionality, diverse data sources used, the sampling plan implemented for the surveys, and the estimation techniques utilized for GHG emissions. Additionally, we outline the methods used to detect agricultural burned area and area under zero-tillage, using remote sensing data. The methodological approach employed to calculate the mean differences in GHG emissions and wheat yield is also discussed. For a detailed methodology section, refer to Supplementary Section 1.

### Verra’s VM0042: methodology for improved agriculture land management

The VM0042 methodology aims to quantify the reduction in GHG emissions achieved by implementing enhanced agricultural land management practices. The interventions could span various practices, encompassing improved nutrient, water, and residue management, modifications to crop planting and harvesting techniques (including agroforestry, crop rotations, and cover crops), as well as adjustments to grazing practices. The projects must comply with multiple applicability conditions in this methodology, encompassing the acceptable data sources for sourcing parameter values for models to estimate emissions, modelling, quantitative adjustments, and land use changes. In our current study, we will focus on the significant and measurable conditions that align with our available datasets from the IGP.

A central requirement outlined in the VM0042 methodology is additionality^15^. It means that GHG emissions reduction would not have happened without the incentive of carbon credits. The VM0042 methodology outlines the steps for additionality tests as follows,

1. *Demonstrate regulatory surplus* In simpler terms, the project should prove that its activities are not mandated by any laws or regulations, both nationally and internationally. For example, if a project aims to reduce emissions by discouraging crop residue burning in an area where it is already legally prohibited, it must prove emissions reductions that surpass the existing regulatory standards to be eligible for carbon credits.
2. *Barrier analysis* The project developers must identify institutional barriers that would prevent the implementation of a change in pre-existing ALM practices to demonstrate additionality.
3. *Common practice analysis* The project developers must demonstrate that the practice change implemented under the project activity is not “common practice”. “Common practice” here means activities adopted across over 20% of the cultivated area. As a result, to demonstrate additionality, any carbon farming project must implement interventions with a pre-existing adoption rate of < 20%. In scenarios where multiple technology interventions (e.g., zero-tillage with improved irrigation water management) are promoted, the weighted mean adoption rate should also fall below 20%. To verify that additionality requirement is met, data can be drawn from censuses, government reports and datasets, peer-reviewed scientific literature, independent research studies, expert surveys, or industry association reports and assessments.

In addition, the project's implementation must not lead to a sustained yield penalty exceeding 5%. The basis for this criterion can be found in peer-reviewed studies or published research on the activity within the region or similar locales. Furthermore, projects incorporating quantitative adjustments (such as reducing fertilizer application rates) must surpass a 5% deviation from the baseline value.

### Household survey data, Punjab

In the first stage of sampling, districts were selected based on the proportion of their net sown area dedicated to coarse (non-basmati) rice varieties and their recent reports of burned areas^25^. Two districts were purposively selected from each category: Ludhiana and Sangrur from the high residue burning group, and Patiala and Fatehgarh Sahib from the low residue burning category. In the second stage, villages were selected based on the availability of Happy Seeders (HSs). HS is a tractor-mounted machine that slices and lifts paddy straw, sows wheat seeds into the soil, and covers the straw over the sown area as mulch. A list of villages with at least one HS user was compiled and 16 villages were randomly chosen from each survey district. In the third stage, farm households were selected from the chosen villages. All households practicing no-till farming were included in the sample, and within the CT user stratum, village-wise random samples were drawn. The total sample size was 1021 farm households across 52 villages, encompassing 561 CT users, 226 ZT drill users, and 234 HS users. A second round of survey was conducted in June–August 2021 among the same set of farmers, and the sample attrition was 19%. The reasons for attrition and the number of replacements due to them were recorded. The input use and output obtained were captured alongside tillage practice adoption of the 2020/21 wheat season in the second round as well.

### Household survey data, Bihar

A multi-level random sampling methodology was used to select districts and Community Development (CD) Blocks, considering the varied agro-ecological sectors and the extent of wheat cultivation areas. 10 districts were selected across 4 agro-ecological zones. From each district, 1 CD Block was included for the study. Each CD Block included 4 villages selected randomly. During February and March 2021, a census was conducted in these villages to identify potential respondents, i.e., the wheat farmers as of the previous season (Rabi 2020/21). Post-census data accumulation and entry, roughly 25 wheat farmers from each village were chosen for interviews. These interviews were conducted from August to September 2021. In the end, data was acquired from 1,003 households covering a diverse array of subjects including socio-demographics, land features, wheat production, varietal preferences, access to seeds, ownership of assets, and technology adoption.

### Key Informant survey data, Bihar

In Bihar, a baseline survey was conducted across its 38 districts, 534 CD Blocks, and 44,874 villages, using the 2011 Census as a sampling frame. The state, divided into four agroecological zones, was studied as part of a project by the Borlaug Institute for South Asia (BISA). A total of 192 treatment villages across all districts and 191 control villages were selected based on specific project criteria and random sampling, excluding uninhabited or small villages (less than 100 households). Two control villages initially selected were replaced due to overlap with intervention villages, resulting in 193 random villages in the final sample. In each village, 2–3 key informants, such as experienced farmers and village heads, were interviewed in 2021 to gather data on demographics, cropping systems, and technology adoption.

### Income data

We obtained monthly income data from the Situation Assessment Survey (SAS) of Agricultural Households, conducted by the National Statistical Office (NSO) in India. The data revealed that, on average, an agricultural household in Bihar earned INR 7542 per month, while in Punjab, the average monthly income was INR 26,701^52^. Assuming a six-month wheat season, an average farmer in Bihar would receive INR 45,252 from wheat cultivation during this period. The potential income from carbon credits, estimated at INR 900 per hectare, would account for approximately 2% of a farmer's total wheat season income.

### Crop residue burning data

We use Sentinel-2 (10 m) multispectral data to accurately estimate agricultural burned area (ABA). We used a locally adapted multi-temporal burned area detection methodology to quantify burned areas after the crop harvest in 2022. Here, a novel satellite data-assisted virtual sampling method was used to collect burned and unburned training samples. Monthly ABAs were extracted at 10 m resolution and were compared with existing global burned area products. On average, the derived ABAs were larger (∼9179 km2 per month) than those reported by global burned area products that are based on MODIS at 500 m and 250 m resolutions. This approach maps ABA from smallholder farms more accurately than other global products, particularly for fields that are smaller than 10 hectares. More details on the methodology to use Sentinel-2 data to detect and quantify ABA in smallholder systems are provided in the paper^53^. The accuracy rate for ABA estimation and zero tillage is 90% and 80% respectively.

### Zero-tillage data

We utilized data provided by IISER, Bhopal researchers who secured Sentinel-2, Level 2A (S2A) product provided by the European Space Agency (ESA). A novel method of modified change detection using early-season high-resolution Sentinel-2 MSI imagery was used to quantify zero-tillage adoption. Ground truth data, including harvesting, sowing dates, and tillage practices, were collected from 426 plots in Punjab, with the classification model achieving 77% accuracy, outperforming existing binary models.

### GHG emissions data

The emission values from crop-related activities specifically from wheat cultivation and rice stubble burning were estimated using the Mitigations Options Tool of the CGIAR Research Program on Climate Change, Agriculture and Food Security (CCAFS-MOT)^54^. A version of this tool was translated to R which was used to process our plot-level dataset. For our study, we focused on plot-level data collected from the Punjab and Bihar household surveys (mentioned above), analyzing 1021 plots from Punjab farmers and 1002 plots from Bihar farmers.

### Estimation of technology effects

Propensity Score Matching (PSM) technique was used to assess the mean difference in GHG emissions and wheat yield between CA adopters and non-adopters. Using a regression model with an adoption dummy or simply comparing mean differences in outcomes, such as yield and GHG emissions, between adopters and non-adopters of CA fails to account for the endogeneity of CA adoption decisions, leading to selection bias. This method may also be affected by observed factors (e.g., education) and unobserved factors (e.g., farmer's risk perception and managerial skills). PSM addresses the endogeneity problem partly in the adoption of CA practices by estimating a single-dimensional score from multiple covariates. This score is used to match adopters and non-adopters of CA based on observed characteristics, reducing bias from observed variables. However, PSM does not account for unobserved heterogeneity, which can still affect the estimation of treatment effects. Despite this limitation, PSM generally provides a more unbiased estimate of the impact than the simple mean difference of outcomes.

We utilize PSM in a two-stage process, first estimating the propensity score through a probit model considering a variety of attributes including plot area; soil attributes such as texture, fertility, and issues of erosion, salinity, or sodicity; water logging concerns; and various management practices like the use of laser land levelers, sowing dates, and tillage methods. Additionally, we considered socio-demographic factors like caste and household size, as well as agronomic practices like mulching and the utilization of both organic and chemical fertilizers. District fixed effects were also incorporated for more granular matching. Then, adopting and non-adopting households are matched based on these scores. This approach ensures comparable groups in terms of observed covariates, aiming to reduce the estimation bias when assessing the Average Treatment Effect (ATE) on crop yields and GHG emissions.

Through PSM, we examine whether CA practices such as zero/reduced tillage, optimal nitrogen fertilizer use, avoidance of stubble burning and optimal irrigation impact yields and GHG emissions. Specifically, we analyze if the adoption of CA incurs a yield penalty while contributing to GHG emissions reduction, thus informing the broader implications of these practices on carbon markets.

### Ethics approvals

This study involved surveying farmers and was conducted in strict accordance with the ethical standards of the CIMMYT Internal Research Ethics Committee (IREC). The protocol for the research was reviewed and approved by the IREC, which is registered with the United States Department of Health and Human Services, Office for Human Research Protections (IORG number: IORG0010747, IRB number: IRB00012744). Informed consent was obtained from all respondents prior to their participation in the study.

### Supplementary Information

Supplementary Information.

## Data Availability

Datasets on district-level crop residue burning in Punjab and zero-tillage adoption in Bihar are included in the supplementary file. Other datasets are available from the corresponding author, Adeeth A. G. Cariappa (a.adeeth@cgiar.org) on reasonable request. The data will be shared in accordance with CIMMYT's data sharing policies and subject to any applicable ethical or confidentiality constraints.
