# Supplementary material for: Evaluating the potential and eligibility of conservation agriculture practices for carbon credits
Source: Sci Rep. 2024 Apr 22;14:9193. doi: 10.1038/s41598-024-59262-6 (PMC11035690; doi:10.1038/s41598-024-59262-6)
Supplement: Supplementary file 1 — Supplementary Information. [file 41598_2024_59262_MOESM1_ESM.docx]

**Supplementary file to Evaluating the Potential and Eligibility of Conservation Agriculture Practices for Carbon Credits**

**Section 1. Study Area and Data Sources**

*Household Surveys*

**Punjab**

In 2021, a household survey was conducted among the same set of farmers who were interviewed in 2018 by International Maize and Wheat Improvement Center (CIMMYT)^1^ with the objective of gaining insights into the input use patterns, impact of tillage technologies, and decision-making processes of farmers regarding the adoption of various non-burning technologies across four districts within the north-western state of Punjab, India. Within Punjab's cropped area of 7.79 million hectares, a substantial portion, specifically 2.92 million hectares (equivalent to 38%), is allocated for paddy cultivation ^2^. Rice farming in this region predominantly falls into two categories: coarse (non-basmati) rice and basmati rice. Basmati rice straw is used as cattle feed, whereas coarse rice straw cannot be used. The latter is more commonly subjected to combine harvesting methods ^3^, resulting in substantial crop residue left in the fields, hence higher burning. Consequently, the difference between the area under basmati rice and the total area under paddy cultivation serves as a proxy for regions susceptible to residue burning ^4^.

The selection of survey districts followed a systematic process. Initially, districts with over 70% of their net sown area dedicated to coarse (non-basmati) rice varieties during the kharif season (July to October) of 2017 were identified^5^. These districts were further categorized into 'high residue burning' and 'low residue burning' based on recent reports of burned areas ^6^. Subsequently, two districts were purposively selected from each category, with geographic proximity as a priority: Ludhiana and Sangrur were chosen from the high residue burning group, while Patiala and Fatehgarh Sahib represented the low residue burning category. All four districts are situated within the South-East Region of the state, minimizing variations in agro-climatic factors. During the kharif season, coarse rice varieties dominate paddy land use in each of the four districts, while wheat takes precedence during the rabi season (November–April). Other significant crops in the region include maize, sugarcane, vegetables, pulses, mustard, and cotton ^7^.

The selection of study villages commenced with the identification of all villages in which at least one individual had purchased a Happy Seeder (HS). A comprehensive list was compiled by combining information from leading HS manufacturers, lists of farmers receiving HS services from Primary Agriculture Cooperative Societies (PACS) and lists of subsidy beneficiaries for HS purchases provided by the State Department of Agriculture. Subsequently, 16 villages were randomly chosen from each survey district using a probability proportionate to the village size approach. Given the relatively low prevalence of ZT wheat in the area, a census survey was initially administered across the 64 selected villages to stratify the sample by wheat establishment method, differentiating between conventional tillage (CT), zero-till (ZT) drill, and HS usage. Based on the data obtained from the census survey, 13 villages were selected from each district, excluding villages with no HS users in 2017. Farm households within the chosen 52 villages (shown in Figure S1) were then categorized into CT, ZT drill, and HS users, based on information from the census survey.

The identification of farm households for the sample survey was carried out with consideration of their relative scarcity. All households practicing no-till farming were included in the sample. Within the CT user stratum, village-wise random samples were drawn using the following selection rule: if the number of no-till households was less than 15, the number of randomly selected CT households was 15 minus the number of no-till households. In cases where the number of no-till households exceeded 15, the number of selected tillage households constituted approximately 25% of the number of no-till households in that village. This approach yielded a total sample size of 1021 farm households across 52 villages, encompassing 561 CT users, 226 ZT drill users, and 234 HS users. Some households employed multiple wheat establishment methods; individuals utilizing the HS (in addition to CT and/or ZT drill) were classified as HS users, while those utilizing a ZT drill (apart from CT) were classified as ZT drill users.

During June-August 2021, a second round of survey was conducted among the same set of farmers. The sample attrition was 19%, and one of the major reasons was farmers leasing out land completely (farmers leaving cultivation). The reasons and the number of replacements due to them are shown in Figure S2. The input use and output obtained were captured alongside tillage practice adoption of 2020/21 wheat season in the second round also.

**Bihar**

The second dataset that we used is from Bihar: a farm-household survey administered by the CIMMYT, focusing on wheat farmers in the state. Following a multi-level random sampling methodology, we purposefully chose districts and Community Development (CD) Blocks, keeping in mind the varied agro-ecological sectors and the extent of wheat cultivation areas. We selected ten districts spread across four agro-ecological zones. Four districts (Paschim Champaran, Purba Champaran, Sheohar and Darbhanga) were sampled from North the Alluvial Plain Zone, two districts (Araria and Madhepura) from the Northeast Alluvial Plain Zone, two districts (Banka and Jamui) from the Southwest Alluvial Plain Zone, and two districts (Nalanda and Patna) from the Southeast Alluvial Plain Zone. From one district, one CD Block was included for the study. Each CD Block included four villages selected randomly. During February and March 2021, we organized a census in these villages to identify potential respondents – that is, the wheat farmers as of the previous season (Rabi 2020/21). The location of these villages on the map of Bihar is shown in Figure S3.

Post census data accumulation and entry, roughly 25 wheat farmers from each village were chosen for interviews. These interviews were conducted from August to September 2021. If any household had relocated during the period between the census and the survey, substitutes were selected from a random farmer list. In the end, we acquired data from 1,003 households covering a diverse array of subjects including socio-demographics, land features, wheat production, varietal preferences, access to seeds, and ownership of assets. The primary outcome variable in this study relates to wheat yields, measured at individual plot levels. Data was collected pertaining to the harvest and size of a randomly chosen wheat plot for the 2020/21 Rabi season, which allowed us to calculate the wheat yields in tons per hectare for that specific plot. In addition, technology adoption and input use, information and market access, livelihood indicators (e.g., food insecurity, dietary diversity etc.) was collected from the household.

**Key Informant survey, Bihar**: Bihar, the third most populous state in India, is administratively divided into 38 districts, 534 CD Blocks (Sub-districts), and 44874 Villages. The Census of 2011, which encompassed all 38 districts and villages, serves as the foundation for the sampling frame. From an agroecological perspective, Bihar is categorized into four zones (AEZ). The key informant interviews were conducted as part of a baseline survey of a project under the Borlaug Institute for South Asia (BISA), Samastipur. For this, 192 treatment villages were deliberately chosen across all Bihar districts, following specific project criteria, ensuring an average of 4-5 villages per district. CD blocks are utilized as the Primary Sampling Unit (PSU) for village selection. The non-intervention/control villages were randomly selected in proportion to the number of treatment villages per CD block. During the control village selection, random sampling was employed, assigning equal probability to all villages in the selected CD block regardless of treatment status, thereby enhancing the random component of sampling. Uninhabited villages and those with fewer than 100 households were excluded from the sampling frame. Subsequently, 191 control villages were randomly chosen across all 38 districts of Bihar.

Two randomly selected control villages were found to be on the list of intervention villages. Replacement villages were randomly selected for these instances. Consequently, the total number of random villages in the sample frame is 193. From each village, 2-3 key informants (e.g., experienced farmers, heads of farmer cooperatives, village heads, etc.) were interviewed, and details about the village, including demographic characteristics, cropping system, technology adoption, etc., were collected in 2021.

**Section 2. Methods**

***Household income***. We utilized monthly income data sourced from the Situation Assessment Survey (SAS) of Agricultural Households, a survey conducted by the National Statistical Office (NSO) under the Ministry of Statistics and Programme Implementation (MoSPI) within the Government of India. This data was collected during the 77th round of the survey, spanning from January 2019 to December 2019, and was referenced to the agricultural year spanning from July 2018 to June 2019.

During this period, the average monthly income per agricultural household in Bihar amounted to INR 7,542, while in Punjab, it was INR 26,701 ^8^. To calculate the income derived from carbon credits during the wheat season as a percentage of the household income, we assumed a wheat season of six months. For example, an average farmer in Bihar would receive an income of INR 900 per hectare in the wheat season. Household income in the wheat season would be INR 45252 (INR 7542 x 6 months). Therefore, an average farmer in Bihar would receive approximately INR 900 from carbon credits equivalent to 2% of their household income in wheat season.

***Detection of zero tillage (ZT)***. To quantify the ZT adoption, we use a novel method of modified change detection that effectively uses the early season high-resolution images from Sentinel-2 MultiSpectral Instrument (MSI). For ground truthing, plot georeferencing was made, and ground truth data was collected for remote sensing of tillage in the plots, after the monsoon harvest in 2022. The data included harvesting dates, sowing dates, as well as information on tillage practices, specifically whether the fields were under conventional tillage (CT), ZT, or other field management practices like the Shallow Tillage (ST), which indicates the use of super seeder. The survey was conducted in a total of 426 plots, with 137 plots practicing CT, 144 plots practicing ST and 145 plots practicing ZT. The data included GPS locations at the four corners and center of each field, which were later used to create the polygons. We overlaid the created polygons on high-resolution imagery in Google Earth for better visualization and manual corrections.

We utilized the Sentinel-2, Level 2A (S2A) product provided by the European Space Agency (ESA), freely available on the GEE platform. The Level 2A data were corrected for surface reflectance and atmospheric conditions using the Sen2Cor algorithm and were provided as 100x100 km² tiles with UTM/WGS84 projection. Our classification model performed better than the existing binary models with a decent accuracy of 77%. Our model performed well when applied over the years, indicating its temporal relevance in differentiating the tillage technologies. Using this approach, we estimated tillage adoption in the state of Punjab for 2020-2022. When compared with village-level survey data of 2022, our model shows less standard deviation (7%). However, the model results are 24% higher than survey estimates, and the model outperforms the survey method on a larger spatiotemporal scale. Overall, our method highlights the importance of early-season satellite imagery and provides a novel approach to detect multiple tillage technologies for better understanding and policy design to propagate sustainable agricultural practices in India.

**GHG estimation**

GHG emissions, confined to the farm-gate were estimated for crop production using a semi-lifecycle approach. GHG emissions caused by tillage and crop establishment, crop management activities such as fertilizer application, and organic inputs like crop residues, manure and compost under specific soil and climatic conditions were accounted for. The plot-level data required for emission estimation includes observations on pedo-climatic characteristics of the plot, yield obtained, fertilizer, manure, compost, and residue added, type of tillage and fuel and energy used within the farm-gate.

The emission values from crop-related activities specifically from wheat cultivation and rice stubble burning were estimated using the Mitigations Options Tool of the CGIAR Research Program on Climate Change, Agriculture and Food Security (CCAFS-MOT) ^10^. It employs a semi-lifecycle approach to estimate GHG emissions using observations of inputs, production, and other management practices at the field level, along with soil and climatic information of each plot. This tool combined multiple empirical models to estimate emissions from various management practices ^11,12^, with a combination of Tier 1 and Tier 2 approaches. GHG emissions are estimated as total Global Warming Potential (GWP) in terms of carbon dioxide equivalent (kg CO2eq) per hectare and as emission intensity in terms of carbon dioxide equivalent (kg CO2eq) per unit of product (kg). A version of this tool was translated to R which was used to process our plot-level dataset. For our study, we focused on plot-level data, analyzing 1,021 plots from Punjab farmers and 1,002 plots from Bihar farmers.

**Propensity Score Matching (PSM)**

Conventional approaches to evaluating the effects of technology adoption often employ a straightforward regression analysis with an adoption dummy and control variables. However, this approach inherently assumes the exogeneity of technology adoption, potentially introducing bias. Such biases may arise from unobserved variables, including farmer's risk perception and managerial skills, both of which could correlate with adoption decisions and resultant outcomes.

To address this endogeneity concern, we adopt the Propensity Score Matching (PSM) technique, a quasi-experimental method that has gained traction in agricultural technology impact assessments ^13–17^. PSM is a valuable statistical approach that can mitigate selection bias by equating groups based on observed covariates influencing treatment assignment, simulating randomization in observational data. Its advantage lies in the reduction of multidimensional covariates into a single-dimensional propensity score, facilitating matching and comparison across treatment groups. However, PSM is limited by its reliance on observed covariates, with any unobserved heterogeneity potentially biasing estimates, and assumes that the treatment effect is constant across different levels of the propensity score.

In the context of our study, it represents the probability that a farm adopts CA given its observed characteristics. The core idea behind PSM is to match treated (farms that adopted CA) and untreated (farms that did not CA) units based on their propensity scores, ensuring that the distribution of observed covariates is similar between the two groups. This ensures that the two groups are comparable on observed characteristics, thereby reducing the bias in estimating the treatment effect ^14^. PSM constructs an artificial control group mitigating the selection bias often associated with non-experimental methods. The fundamental assumption behind PSM is the Conditional Independence Assumption (CIA), which states that, conditional on the propensity score, the potential outcomes are independent of treatment assignment. This implies that, given the propensity score, the distribution of observed covariates will be similar between treated and untreated farms ^18^.

PSM was used to assess the differences in GHG emissions and yield between CA adopters and non-adopters. In the first stage, we estimated the propensity score using a probit model, incorporating a range of attributes such as plot area; soil attributes such as texture, fertility, and issues of erosion, salinity, or sodicity; water logging concerns; and various management practices like the use of laser land levelers, sowing dates, and tillage methods. Additionally, we considered socio-demographic factors like caste and household size, as well as agronomic practices like mulching and the utilization of both organic and chemical fertilizers. District fixed effects were also incorporated for more granular matching. In the second stage, these scores were used to match CA adopting and non-adopting households based on their propensity scores.

After matching, we compare the average crop yields between the treated and untreated groups to estimate the Average Treatment Effect (ATE). The difference in average yields provides a less biased estimate of the impact of CA on crop yields. The primary objective was to assess yield outcomes, specifically examining whether there was any yield penalty and GHG emissions reduction potential associated with the adoption of conservation agriculture practices such as zero/reduced tillage, optimum nitrogen fertilizer use, and refraining from stubble burning.

**References**

1. Keil, A. *et al.* Changing agricultural stubble burning practices in the Indo-Gangetic plains: is the Happy Seeder a profitable alternative? *Int J Agric Sustain* **19**, 128–151 (2021).

2. GoI. *All India report on agriculture census 2010–11*. https://agcensus.nic.in/document/ac1011/reports/air2010-11complete.pdf (2015).

3. Gupta, R. *Causes of emissions from agricultural residue burning in north-west India: Evaluation of a technology policy response*. https://ideas.repec.org/p/snd/wpaper/66.html (2012).

4. Lohan, S. K. *et al.* Burning issues of paddy residue management in north-west states of India. *Renewable and Sustainable Energy Reviews* **81**, 693–706 (2018).

5. APEDA. *Basmati survey report, Vol. 1*. https://apeda.gov.in/apedawebsite/six_head_product/BSK-2017/Basmati_Report-1.pdf (2017).

6. Kaur, A. & Rani, J. An approach to detect stubble burned areas in Punjab by digitally analyzing satellite images. *Journal for Research* **2**, 64–69 (2016).

7. GoP. Department of Agriculture & Farmer Welfare. https://agripb.gov.in (2019).

8. Ministry of Agriculture & Farmers Welfare. Income of Farmers. *NSS Report No. 587: Situation Assessment of Agricultural Households and Land and Livestock Holding of Households in Rural India, 2019* https://pib.gov.in/PressReleasePage.aspx?PRID=1884228 (2022).

9. Deshpande, M. V., Pillai, D. & Jain, M. Agricultural burned area detection using an integrated approach utilizing multi spectral instrument based fire and vegetation indices from Sentinel-2 satellite. *MethodsX* **9**, 101741 (2022).

10. Feliciano, D. A review on the contribution of crop diversification to Sustainable Development Goal 1 “No poverty” in different world regions. *Sustainable Development* **27**, 795–808 (2019).

11. Bouwman, A. F., Boumans, L. J. M. & Batjes, N. H. Emissions of N2O and NO from fertilized fields: Summary of available measurement data. *Global Biogeochem Cycles* **16**, 6-1-6–13 (2002).

12. Yan, X., Yagi, K., Akiyama, H. & Akimoto, H. Statistical analysis of the major variables controlling methane emission from rice fields. *Glob Chang Biol* **11**, 1131–1141 (2005).

13. Rosenbaum, P. R. & Rubin, D. B. The central role of the propensity score in observational studies for causal effects. *Biometrika* **70**, 41–55 (1983).

14. Dehejia, R. H. & Wahba, S. Causal Effects in Nonexperimental Studies: Reevaluating the Evaluation of Training Programs. *J Am Stat Assoc* **94**, 1053–1062 (1999).

15. Mishra, A. K., Khanal, A. R. & Mohanty, S. Gender differentials in farming efficiency and profits: The case of rice production in the Philippines. *Land use policy* **63**, 461–469 (2017).

16. Mason, N. M., Wineman, A., Kirimi, L. & Mather, D. The Effects of Kenya’s ‘Smarter’ Input Subsidy Programme on Smallholder Behaviour and Incomes: Do Different Quasi-experimental Approaches Lead to the Same Conclusions? *J Agric Econ* **68**, 45–69 (2017).

17. Paudel, G. P., Krishna, V. V. & McDonald, A. J. Apparent Gains, Hidden Costs: Examining Adoption Drivers, Yield, and Profitability Outcomes of Rotavator Tillage in Wheat Systems in Nepal. *J Agric Econ* **71**, 199–218 (2020).

18. Cunningham, S. Matching and Subclassification. in *Causal Inference: The Mixtape* (2021).

**Figure and Table Legends**

[**Supplementary Figure 1**. Map of Punjab showing sample villages.](#_Toc150949516)

[**Supplementary Figure 2**. Reasons for sample attrition in the household survey (2021)](#_Toc150949517)

[**Supplementary Figure 3**. Map of Bihar Showing the Sample Villages](#_Toc150949518)

[**Supplementary Table 1.** Area under crop residue burning during 2022 in Punjab.](#_Toc160707638)

[**Supplementary Table 2**. District-wise adoption of zero-tillage in Bihar](#_Toc160707639)

[**Supplementary Table 3.** Change in carbon credits generated and its value when residue burning is excluded in Punjab.](#_Toc160707640)

[**Supplementary Table 4.** Deviation of farmers' fertilizer use from the recommended dose of application in Punjab.](#_Toc160707641)

[**Supplementary Table 5**. Description of variables used in various PSM models](#_Toc160707642)

**
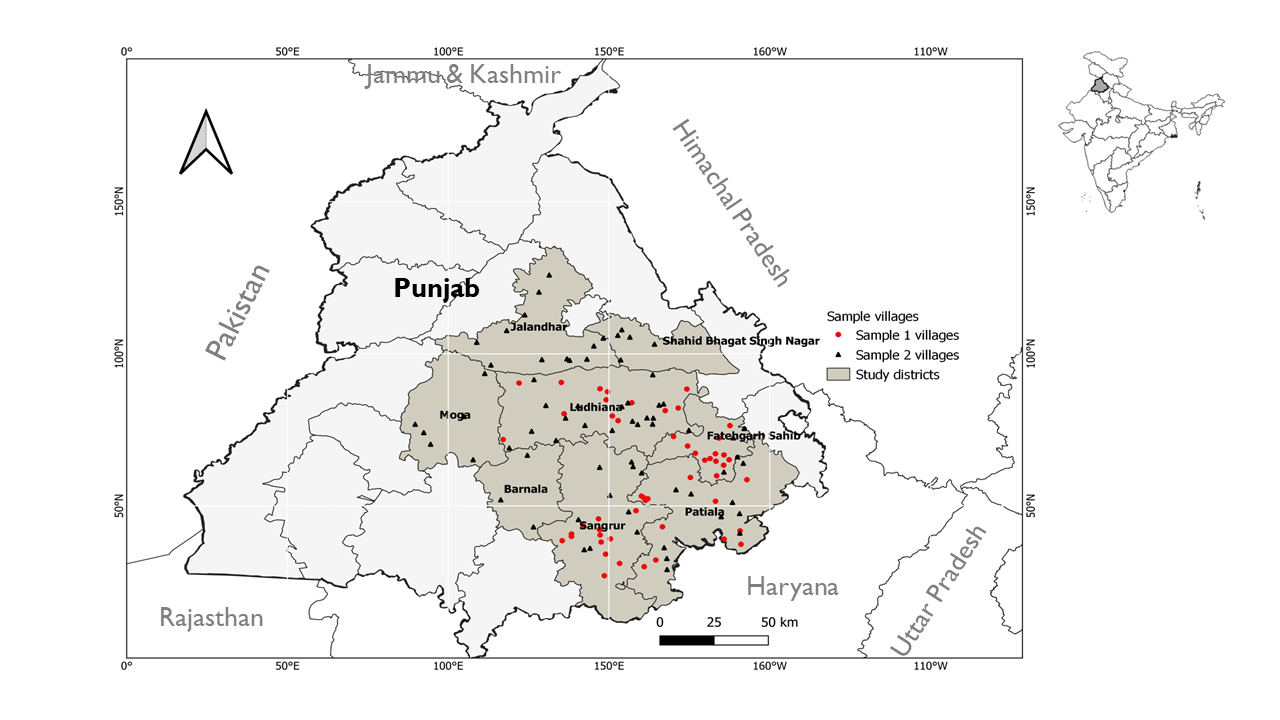
Supplementary Figure 1**. Map of Punjab showing sample villages.


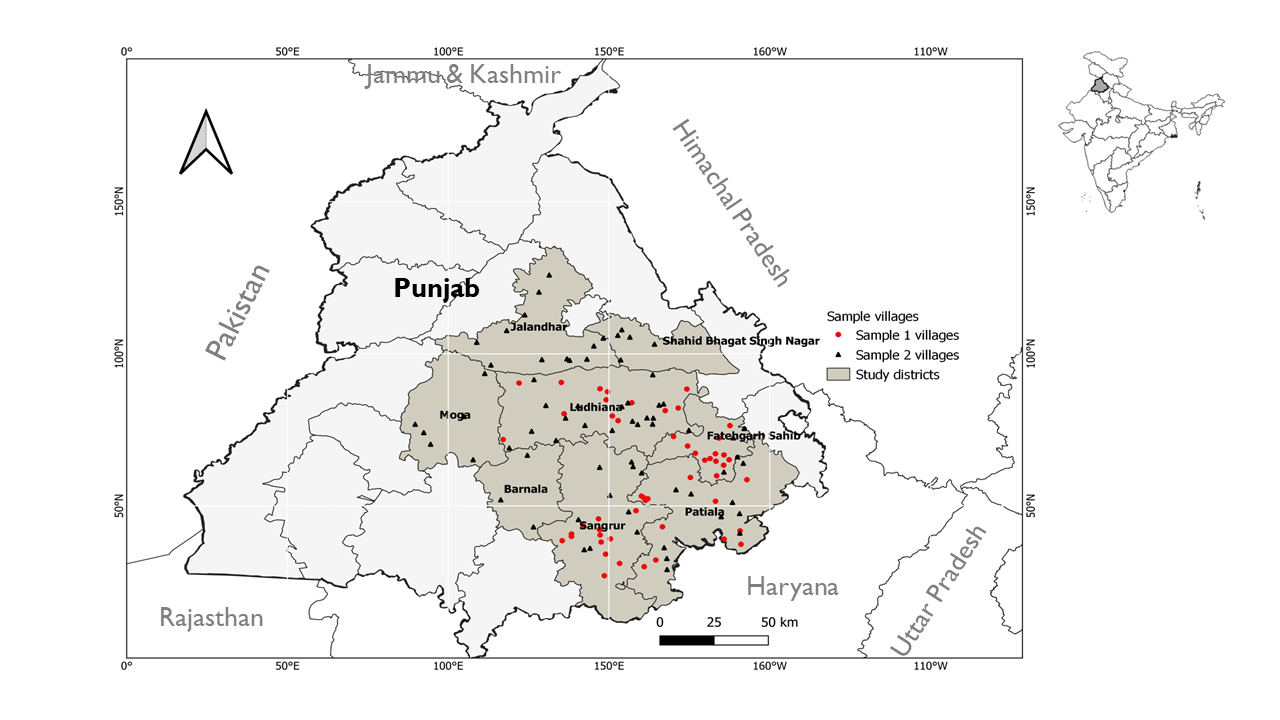


*Note*: Red dots indicate the location of sample villages. Black dots indicate the location of villages where village surveys only were conducted.

Map was created using QGIS Desktop 3.30.3, a free and open-source Geographic Information System, available at: <https://qgis.org>. The base maps were derived from shapefiles obtained from the open source Administrative Boundary Database of the Survey of India (<https://onlinemaps.surveyofindia.gov.in/Digital_Product_Show.aspx>).

**Supplementary Figure 2**. Reasons for sample attrition in the household survey (2021)

*Note*: HHH: Household head; HH: Household.

Figures show the number of households replaced due to this reason.

**Supplementary Figure 3**. Map of Bihar Showing the Sample Villages


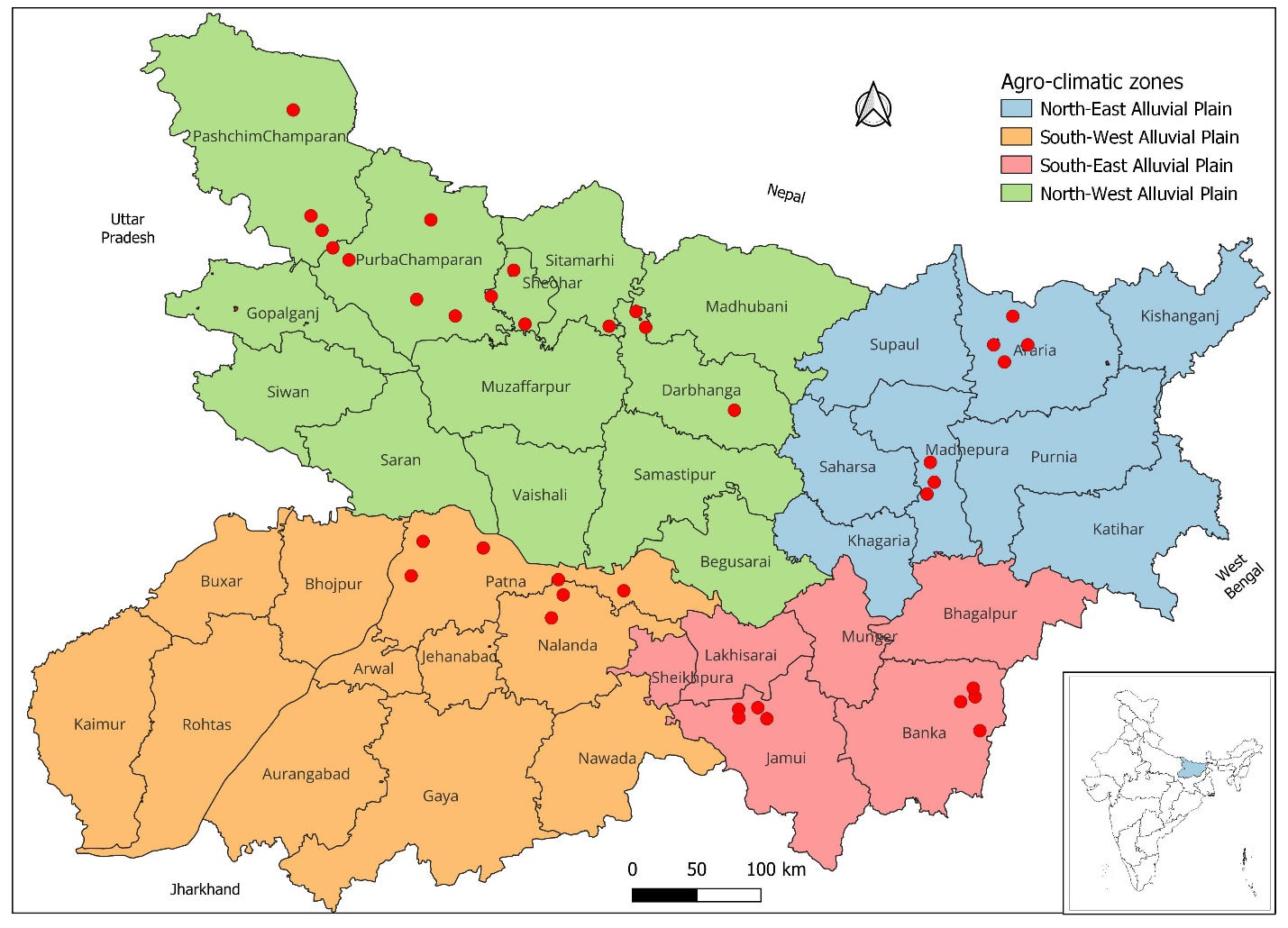
*Note*: Circles in the map show the location of study villages.

Map was created using QGIS Desktop 3.30.3, a free and open-source Geographic Information System, available at: <https://qgis.org>. The base maps were derived from shapefiles obtained from the open source Administrative Boundary Database of the Survey of India (<https://onlinemaps.surveyofindia.gov.in/Digital_Product_Show.aspx>).

**Datasets**

**Supplementary Table 1.** Area under crop residue burning during 2022 in Punjab.

| **District** | **Agriculture Burned Area**  **(% of total cultivated area)** |
| --- | --- |
| Amritsar | 57 |
| Barnala | 61 |
| Bathinda | 49 |
| Faridkot | 60 |
| Fatehgarh Sahib | 66 |
| Fazilka | 31 |
| Firozepur | 59 |
| Gurdaspur | 39 |
| Hoshiarpur | 20 |
| Jalandhar | 42 |
| Kapurthala | 44 |
| Ludhiana | 46 |
| Mansa | 53 |
| Moga | 61 |
| Muktsar | 48 |
| Nawanshahr | 28 |
| Pathankot | 15 |
| Patiala | 53 |
| Rupnagar | 23 |
| Sas Nagar | 13 |
| Sangrur | 62 |
| Tarn Taran | 56 |
| *Punjab* | *46* |

*Source*: Authors estimation based on remote sensing data

**Supplementary Table 2**. District-wise adoption of zero-tillage in Bihar

| **District** | **Share of wheat area under Zero-tillage (%)** |
| --- | --- |
| Araria | 03 |
| Arwal | 10 |
| Aurangabad | 30 |
| Banka | 20 |
| Begusarai | 10 |
| Bhagalpur | 05 |
| Bhojpur | 30 |
| Darbhanga | 5 |
| Gaya | 10 |
| Gopalganj | 65 |
| Jamui | 12 |
| Jehanabad | 10 |
| Khagaria | 10 |
| Lakhisarai | 20 |
| Madhepura | 07 |
| Madhubani | 18 |
| Munger | 10 |
| Muzaffarpur | 15 |
| Nalanda | 20 |
| Nawada | 20 |
| Pashchim Champar | 10 |
| Patna | 15 |
| Purba Champaran | 23 |
| Purnia | 02 |
| Rohtas | 20 |
| Saharsa | 05 |
| Samastipur | 10 |
| Saran | 10 |
| Sheikhpura | 10 |
| Sheohar | 06 |
| Sitamarhi | 10 |
| Supaul | 08 |
| Vaishali | 13 |
| *Bihar* | *14* |

*Source*: CIMMYT Key Informant Survey 2021

**Supplementary Table 3.** Change in carbon credits generated and its value when residue burning is excluded in Punjab.

|  | **Punjab**  **(with residue burning)** | **Punjab**  **(without residue burning)** |
| --- | --- | --- |
| Carbon credits generated (numbers) | 1.97 | 1.07 |
| Value of carbon credits per hectare @ USD 25 per credit | INR 3,873  (~USD 49) | INR 2,098  (~USD 27) |
| 60% per-hectare of carbon credits value which reaches farmer in wheat season | INR 2,324  (~USD 30) | INR 1,259  (~USD 16) |
| Additional revenue for a typical farmer (cultivating in 0.6 ha in Bihar and 4.9 ha in Punjab) | INR 11,386  (~USD 145) | INR 6169  (~USD 78) |
| Revenue from carbon credits as % of yearly income | 7 | 4 |

*Note*: All the notes of Table 1 apply here.

**Supplementary Table 4.** Deviation of farmers' fertilizer use from the recommended dose of application in Punjab.

| **District** | **PAU recommended dose of application (kg/ha)** | | **Farmers application (kg/ha)** | | **Deviation in fertilizer application (%)** | |
| --- | --- | --- | --- | --- | --- | --- |
|  | **Urea** | **DAP** | **Urea** | **DAP** | **Urea** | **DAP** |
| Fatehgarh Sahib | 271.70 | 135.85 | 314.72 | 235.26 | 15.83 | 73.17 |
| Ludhiana | 271.70 | 135.85 | 273.36 | 214.32 | 0.61 | 57.76 |
| Patiala | 271.70 | 135.85 | 330.66 | 236.68 | 21.70 | 74.22 |
| Sangrur | 271.70 | 135.85 | 341.27 | 233.97 | 25.60 | 72.23 |
| *Punjab* | *271.70* | *135.85* | *315.00* | *230.06* | *15.94* | *69.35* |

**Supplementary Table 5**. Description of variables used in various PSM models.

| **Sl. No.** | **Variable** | **Variable description** |
| --- | --- | --- |
| Dependent variables | | |
| 1 | Yield | Natural logarithm of wheat productivity |
| 2 | GHG | GHG emissions in kgCO_2_e per hectare |
| CA practices evaluated | | |
| 1 | Zero tillage | Plots where wheat seeds are sown directly without tillage. Variable takes value 1 if farmers practice zero tillage and 0 otherwise. |
| 2 | Reduced tillage | Plots where wheat seeds are sown after 1-2 rounds of tillage. Variable takes value 1 if farmers practice reduced tillage and 0 otherwise |
| 3 | No residue burning | Variable takes value 1 if farmers burn paddy crop in Kharif season and 0 otherwise. |
| 4 | No N overuse | Variable takes value 1 if farmers apply N more than the recommended dose of 120kg/ha in Bihar and 123.5 kg/ha in Punjab, and 0 otherwise. |
| 5 | Reduced irrigation | Variable take value 1 if farmers apply >3 irrigations and 0 otherwise. |
| Other observed household and plot characteristics | | |
| 1 | Age | Age of the household head in years |
| 2 | Education | Years of education of household head |
| 3 | Caste group | Variable takes values,  1 = SC, 2 = ST, 3 = OBC, 4 = General category, and 99 = Can’t tell/Don’t know |
| 4 | Household size | Number of adult members in the household |
| 5 | Owned land | Land owned by farmers in hectares |
| 6 | Leased-in land | Land leased-in by farmers in hectares |
| 7 | Slope | 1 = leveled field; 2 = gentle slope; 2 = steep slope |
| 8 | Soil type | 1 = clayey, 2 = loamy; 3 = sandy |
| 9 | Soil fertility | 1 = low fertile; 2 = medium fertile; 3 = high fertile |
| 10 | Water logging | Farmers had water logging problems in the plot (at least in some parts) (1= yes/ 0 = no) |
| 11 | Soil salinity or sodicity | Farmers had soil salinity or sodicity problems in the plot (at least in some parts) (1= yes/ 0 = no) |
| 12 | Soil erosion | Farmers had soil erosion problems in the plot (at least in some parts) (1= yes/ 0 = no) |
| 13 | LLL | Variable take a value of 1 if farmers used laser assisted land leveler and 0 otherwise |
| 14 | Cattle | Number of cattle owned |
| 15 | Buffalo | Number of buffaloes owned |
| 16 | Manure | Variable takes a value of 1 if farmers use animal manure (cow dung/farmyard manure) and 0 otherwise |
| 17 | Mulching | Variable takes a value of 1 if farmers use crop residues as mulch and 0 otherwise |
| 18 | Early sowing | Wheat sowing in October (early sowing) (1= yes/ 0 = no) |
